# Pretreatment Hepatoprotective Effect of the Marine Fungus Derived from Sponge on Hepatic Toxicity Induced by Heavy Metals in Rats

**DOI:** 10.1155/2013/510879

**Published:** 2013-01-13

**Authors:** Nehad M. Abdel-Monem, Ahmed M. Abdel-Azeem, El-Sayed H. El-Ashry, Doaa A. Ghareeb, Asmaa Nabil-adam

**Affiliations:** ^1^Department of Biochemistry, Faculty of Science, Alexandria University, Alexandria, Egypt; ^2^Laboratory of Systemic Mycology, Department of Botany, Faculty of Science, Suez Canal University, Ismaileya, Egypt; ^3^Department of Organic Chemistry, Faculty of Science, Alexandria University, Alexandria, Egypt; ^4^Marine Chemistry Division, National Institute of Oceanography and Fisheries (NIOF), Marine Biotechnology and Natural Product Lab, Alexandria, Egypt

## Abstract

The aim of this study was to evaluate the pretreatment hepatoprotective effect of the extract of marine-derived fungus *Trichurus spiralis* Hasselbr (TS) isolated from *Hippospongia communis* sponge on hepatotoxicity. Twenty-eight male Sprague-Dawley rats were divided into four groups (*n* = 7). Group I served as −ve control, group II served as the induced group receiving subcutaneously for seven days 0.25 mg heavy metal mixtures, group III received (i.p.) TS extract of dose 40 mg for seven days, and group IV served as the protected group pretreated with TS extract for seven days as a protection dose, and then treated with the heavy metal-mixture. The main pathological changes within the liver after heavy-metal mixtures administrations marked hepatic damage evidenced by foci of lobular necrosis with neutrophilic infiltration, adjacent to dysplastic hepatocytes. ALT and AST measurements show a significant increase in group II by 46.20% and 45.12%, respectively. Total protein, elevated by about 38.9% in induction group compared to the −ve control group, in contrast to albumin, decreased as a consequence of metal administration with significant elevation on bilirubin level. The results prove that TS extract possesses a hepatoprotective property due to its proven antioxidant and free-radical scavenging properties.

## 1. Introduction

### 1.1. Heavy Metals as Major Toxicological Problems

A number of trace metals are used by living organisms to stabilize protein structures, facilitate electron transfer reactions, and catalyze enzymatic reactions [1]. For example, copper (Cu), zinc (Zn), and iron (Fe) are essential as constituents of the catalytic sites of several enzymes [2]. However, other metals, such as lead (Pb), mercury (Hg), and cadmium (Cd) may displace or substitute for essential trace metals and interfere with proper functioning of enzymes and associated cofactors. Metals are usually present at low (or very low) concentrations in the oceans [1]. In coastal waters, metals can occur at much higher concentrations, probably due to inputs from river systems [3]. Close to urban centers, metal pollution has been associated with sewage outlets [4, 5].

Metal-induced toxicity is very well reported in the literature [6, 7]. One of the major mechanisms behind heavy metal toxicity has been attributed to oxidative stress. A growing amount of data provide evidence that metals are capable of interacting with nuclear proteins and DNA, causing oxidative deterioration of biological macromolecules [6, 7]. One of the best evidence supporting this hypothesis is provided by the wide spectrum of nucleobase products, typical for the oxygen attack on DNA in cultured cells and animals [7, 8].

Cadmium (Cd) is listed by the US Environmental Protection Agency as one of 126 priority pollutants. The most dangerous characteristic of cadmium is that it accumulates throughout a lifetime. Cadmium accumulates mostly in the liver and kidney, and has a long biological half-life time of 17 to 30 years in humans [9, 10].

Lead is known to induce a broad range of physiological, biochemical, and behavioral dysfunctions in laboratory for animals and humans [11, 12], including central and peripheral nervous systems [13], hemopoietic system [14], cardiovascular system [15], kidneys [16], liver [17], male and female reproductive systems [18, 19].

Mercury is a transition metal. It promotes the formation of reactive oxygen species (ROS) such as hydrogen peroxides. These ROS enhance the peroxides and reactive hydroxyl radicals [20, 21]. Mercuric chloride is an inorganic compound that is used in agriculture as a fungicide in medicine as topical antiseptic and disinfectant and in chemistry as an intermediate in the production of other mercury compounds [22]. Poisoning from environmental sources usually arises from contaminated drinking water as well as plant and animal sourced food products. The metal has been reported to be highly prone to bioaccumulation, leading to biomagnification along the food chain [23]. The absorption, distribution, metabolism, excretion, and toxic dynamics of mercury have been reported to depend on the form and oxidation states [23]. The forms of mercury which are important from a toxicological point of view are elemental (vapor), inorganic salts, and organic salts of mercury. Ingestion of inorganic mercury salts such as mercuric chloride had been reported to cause mainly severe gastrointestinal irritation and renal failure [24]. The toxic effects of an organic and elemental mercury have also been widely reported [25]. Several epidemiologic studies had been conducted on the exposure of humans to mercury through fish and marine mammals' consumption in different geographical areas [26].

Cobalt and nickel are essential trace metals in the human diet. Also, they are major components of the alloys employed in the plate and screw used for connecting bones in orthopedic surgery and in the manufacture of artificial organs [27].

 Cobalt is also used as coloring agents for pottery, ceramics, and glass. However, excessive amounts of these transitional metal ions are toxic. For example, cobalt and nickel salts have been reported to induce convulsions [28]; and to cause DNA strand breaks [29]; and to be organ toxic [30]. Cobalt salts are thought to promote the oxidation of reduced glutathione [31] to produce the reduction on a number of hepatic hem proteins such as cytochrome P450, and to interfere with heme metabolism by accelerating its breakdown and inhibiting its synthesis [32]. In addition, numerous authors have studied the impact of nickel on health. Nickel can cause dermatitis to certain persons [33]. Particles of nickel may cause some morphological transformations in numerous cellular systems and chromosomal aberrations [34]. Cobalt was also found obviously harmful on the prenatal development of mice, rats, and rabbits [35].

Nickel breaks down the immunity by affecting the T-cell system and suppresses the activity of natural killer cells in rats and mice [36, 37]. Nickel has been shown to interact with a number of trace elements that include iron, zinc, copper, manganese, sodium, and potassium [38, 39]. Nickel mobilizes and promotes the excretion of copper, zinc, and manganese from organs and promotes storage of chromium in organs [40]. 

The salts of nickel as particles of nickel can be allergens and carcinogens in man while forming the oxygenated radicals [41]. This cytotoxicity was investigated in numerous microorganisms [42]. Nickel was also found to be responsible for many sexual disorders [43].

### 1.2. Marine Natural Product as Potent Detoxifier Agent

Over the last forty years, sponge (phylum Porifera) has been identified as an excellent source of unique marine natural products, having a high incidence of biologically active compounds than any single marine phylum [44]. The exploration of microorganisms living inside invertebrates is one of the most exciting strategies to solve the pressing supply issue inherent to marine drug discovery. Marine microorganisms, including fungi, have shown to be the potential source of pharmacologically active metabolites, because of their capability to adapt and survive in the marine environment, and to produce unique secondary metabolites [45].

Fungi are known to tolerate and detoxify metals by several mechanisms, including valence transformation, extra- and intracellular precipitation, and active uptake [46, 47]. The high surface to the volume ratio of microorganisms and their ability to detoxify metals are among the reasons that they are considered as a potential alternative to synthetic resins for remediation of dilute solutions of metals and solid wastes [48, 49]. 

Metal resistance is defined as the ability of an organism to survive metal toxicity by means of a mechanism produced in direct response to metal species concerned. Biological mechanisms implicated in fungal survival include extracellular precipitation, complexion and crystallization, transformation of metals, biosorption to cell wall and pigments, decreased transport or impermeability, efflux, intracellular compartmentation, and sequestration [47, 50–54].

## 2. Materials and Methods

### 2.1. Materials

#### 2.1.1. Sampling

Samples of honeycomb sponge (*Hippospongia communis*) were collected from the Egyptian western region of the Mediterranean Sea (from Sidi-Krir to El-Salloum) by dragging ships.

#### 2.1.2. Isolation of Sponge-Derived Fungi

To get rid of nonspecific fungal propagules from seawater column on sponge and jellyfish surfaces, animal tissues were rinsed three times with sterile seawater. The surface of the sample was disinfected with 70% ethanol for 2 minutes. The inner tissue was taken out with a scalpel and forceps and then cut into small cubes approx. 0.5 cm^3^. A total of 15–20 cubes of each sample were placed on isolation media.

All isolation and culture maintaining media for marine taxa were prepared by sea water (SW) and isolation media basically were supplemented with Rose bengal (1/15,000) and chloramphenicol (50 ppm) for suppression of bacterial growth. Five media were adopted for isolation after Atlas (2004) they were: Sea Water Rose bengal Chloramphenicol Agar (SWRCA), Sea Water Czapeks Yeast Extract Agar (SWCYA), Sea Water Oatmeal agar (SWOA), Sea Water Agar (SWA), and Sea Water Potato Dextrose Agar (SWPDA).

For maintaining cultures and for proper identification, pure cultures of isolated fungi were grown on standard media such as Vegetable Agar (V8), Oatmeal Agar (OA), Malt Extract Agar (MEA), Potato Dextrose Agar (PDA), and Potato Carrot Agar (PCA).

#### 2.1.3. Identification of Isolates

Taxonomic identification using morphology characteristics of fungal isolates down to the species level on standard media was mainly based on the following identification keys: Raper and Thom [55], Pitt [56] for *Penicillium* (on Czapek Yeast Extract Agar (CYA) and Malt-Extract Agar (MEA)); Raper and Fennell [57] for *Aspergillus* (on Czapek Agar (CZ)); Ellis [58, 59] for dematiaceous hyphomycetes (Potato Carrot Agar (PCA)); Booth [60] for *Fusarium* (Potato Dextrose Agar (PDA)); von Arx [61], Domsch et al. [62], Watanabe [63] for miscellaneous fungi (on MEA, PDA, CYA); von Arx et al. [64] and Cannon [65] for *Chaetomium* (Oat Meal Agar + Lupin Stem (OA + LUP)). The systematic arrangement follows the latest system of classification appearing in the 10th edition of Ainsworth and Bisby's Dictionary of the fungi [66] and Species Fungorum website (http://www.speciesfungorum.org/Names/Names.asp).

### 2.2. Preparation of Marine Fungus Extract

**Figure 1:**
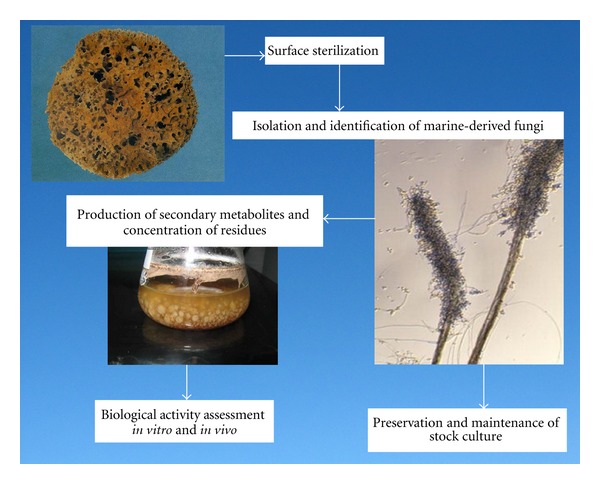
Schematic overview on important steps involved in the isolation of fungi from marine sponge and in preparation of their secondary metabolites.

Regarding fungal diversity of *Hippospongia communis* a total number of 18 taxa were encountered which only one ascosporic species were recorded. *Trichurus spiralis* Hasselbr. has been selected as a promising taxon for *in vitro* and *in vivo* biochemical assays. Preparative-scale production (0.5 L) was carried out in 1 L Erlenmeyer flasks contained potato dextrose extract (Difco) for 2 weeks at 28°C in a shaking incubator at 102 rpm. Pellets were homogenized and centrifuged by using cooling centrifuge at 8000 rpm for 2 min at 4°C. Resultant mixtures were extracted with ethyl acetate (1 × 50 mL), the organic fractions were combined, and the solvent removed at reduced pressure and 35°C. Residues were redissolved in DMSO for further bioassay. The steps of isolation and extraction of fungi and secondary metabolites are shown in Figure 1. 

### 2.3. Animals and Treatment

A total number of 28 adults white male, Sprague Dawley rats, weighing from 100 to 120 g, were obtained from the animal house of the Faculty of Veterinary Medicine, Assiut University. They were kept in plastic cages, each cage containing five animals. They were maintained under standard laboratory conditions of temperature (about 33 ± 3°C), humidity (20 ± 2%), and duration of light (7:00 a.m. to 7:00 p.m.)/dark (7:00 p.m. to 7:00 a.m.) cycles and were fed on standard rodent chow, with water provided ad libitum. After 1 week of acclimatization to the laboratory environment, the rats were divided into the following groups:

 Group I: received saline solution subcutaneously for one week and served as the negative (–ve) control group.
 Group II: received subcutaneously for one week; 0.25 mg /100 gm body weight/day of the heavy metal mixture (Ni, Cd, Co and Hg chloride and Pb acetate) and served as an induced toxicity group.
 Group III: received intraperitoneal (i.p); 40 mg/100 g/body weight/day, of Trichurus spiralis extract (the most effective fungal extract) dose for one week and served as a positive (+ve) control group.
 Group IV: received i.p dose of Trichurus spiralis extract as in group III (40 mg/100 g/body weight/day), for one week as a protection dose before administration of heavy metal mixture (Ni, Cd, Co and Hg chloride and Pb acetate) dose as mentioned in group III. This group served as a protective group.

### 2.4. Biochemical Profiling for Fungus Extract

#### 2.4.1. Elemental Analysis of *Trichurus spiralis* Extract

The fungal extract subjected to elemental analysis instruments to determine hydrogen, carbon, nitrogen, and sulfur ratio (Elemental analysis CHNS elementary, Vario EL III, Germany).

#### 2.4.2. Determination of Total Phenolic Content in (T.S) Fungus Extract

Total phenolic compounds in the fungal extract were determined by the method of [67].

#### 2.4.3. Determination of Total Flavonoid Content in (T.S) Fungus Extract

Total flavonoid content was determined by a colorimetric method of [68].

#### 2.4.4. Diphenyle-*α*-Picrylhydrazyl (DPPH) Radical Scavenging Effect of (T.S) Fungus Extract

DPPH radical scavenging assay of the total extract was performed by using the previously established modified methodology of [69, 70]:

(1) $$\begin{array}{c} \%\;\;\text{scavenging}=\frac{\left[A_{\text{control}}-A_{\text{sample}}\right]}{A\text{control}\times 100}. \end{array}$$

#### 2.4.5. Determination of *Thiobarbituric* Acid Reactive Substance Method Using TBARS Assay for (T.S) Fungus Extract

The method used was adapted from [71] and modified by K.M. Fisch:

(2) $$\begin{array}{c} \%\;\;\text{scavenging}=\frac{\left[A_{\text{control}}-A_{\text{sample}}\right]}{A\;\text{control}\times 100}. \end{array}$$

### 2.5. Biochemical Assay in Serum

The appropriate kits (Bio diagnostic kits) were used for the determination of serum total protein according to [72], aminotransferase activities of aspartate aminotransferase (AST) and alanine aminotransferase (ALT) according to [73]. Determination of albumin level was determined according to the method of [74]. Determination of total bilirubin was determined by the method described by [75].

### 2.6. Histopathology

The fixed liver tissues in formalin were dehydrated in ascending grades of alcohol, then cleaned by immersing the tissues in xylene for 1 h (three times), followed by impregnation in melted paraffin, in wax, then in oven at 60°C for 1 h. The specimens were embedded in paraffin and were left to solidify at RT. Using a rotatory microtone, sections of 5 *μ*m thick were cut and mounted on clean glass slides. Sections were stained with hematoxylin and eosin (H&E) and examined for any histopathological changes [76]. 

### 2.7. Statistical Analysis

The data were given as individual values and as means (X) ± standard deviation (SD) for 7 animals in each group. Comparisons between the means of various treatment groups were analyzed using least significant difference (LSD) test. Differences were considered significant at *P* < 0.05. All statistical analyses were performed using the statistical software SPSS, version 11.5.

## 3. Result 

**Figure 2:**
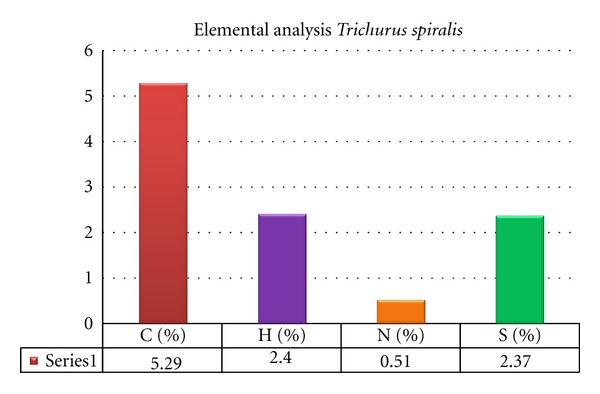
The elemental analysis of *Trichurus spiralis* extract as percentages.

**Figure 3:**
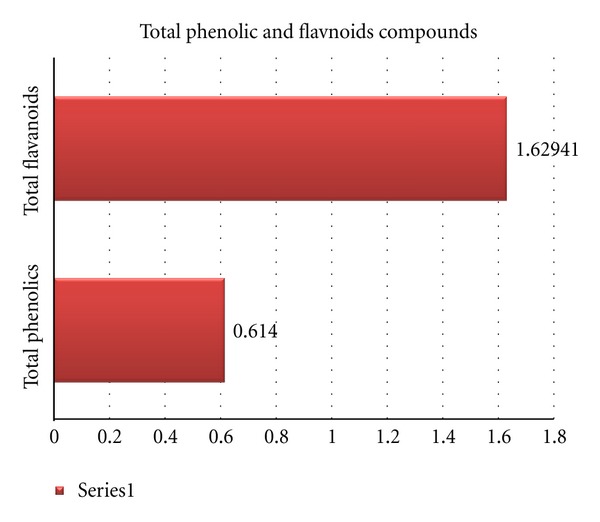
Total phenolic and flavnoids content of *Trichurus spiralis* extract.

**Figure 4:**
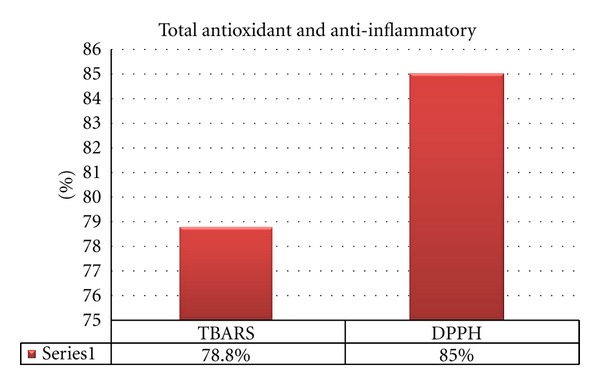
Total antioxidant capacity of *Trichurus spiralis* extract (6 mg/mL). (The percent of *Trichurus spiralis* inhibition to word oxidative stress and lipid peroxidation *in vitro*).

The biochemical profile for *Trichurus spiralis* fungus extract show the higher ratio of sulfur content in the elemental analysis as shown in Figure 2, where the flavnoids content in fungal extract is higher than phenolic as shown in Figure 3. The antioxidant capacity using DPPH assay and inhibition of lipid peroxidation using TABRS *in vitro* show higher antioxidant capacity by 85% and 78.80%, respectively, as shown in Figure 4.

### 3.1. Mortality Rate

**Figure 5:**
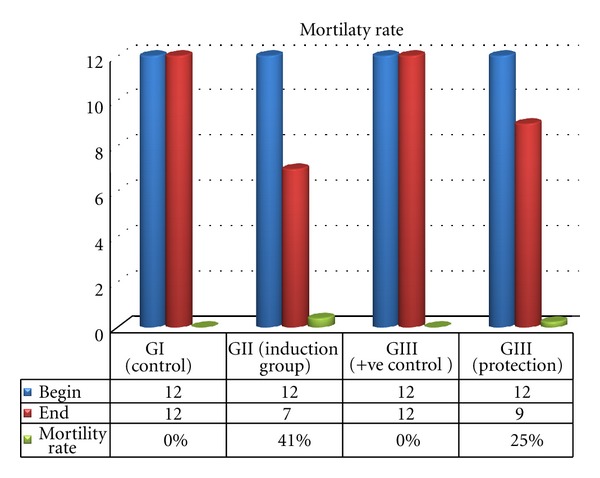
Mortality rat for each group during the study course.

The courses of mortality rate for each group are shown in Figure 5. In the group II (induction group), four of 12 animals died by day 6: two of them died within the first 48 h, and the two others died by 72 and 144 h following induction of heavy metal mixtures. In the group IV (protection group), two rats died in first 72 h (after marine fungal extract and heavy metal administration), where the third died by 120 h. although, there were no differences at the other two groups, group I (−ve control) and group III (+ve control).

#### 3.1.1. Histological Findings

**Figure 6:**
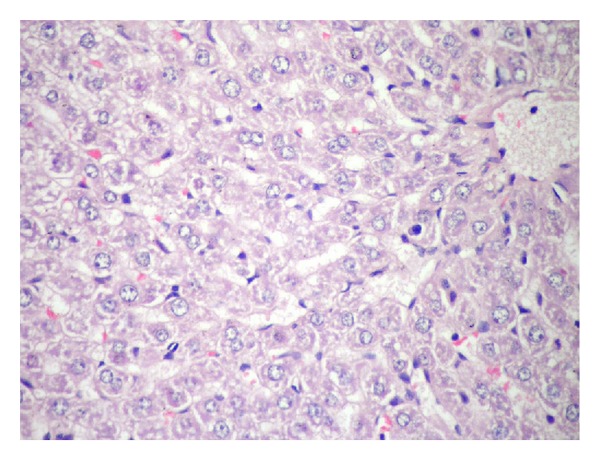
The normal liver showing hepatic architecture formed of cords of hepatocytes separated by hepatic sinusoids (H&E 400x).

**Figure 7:**
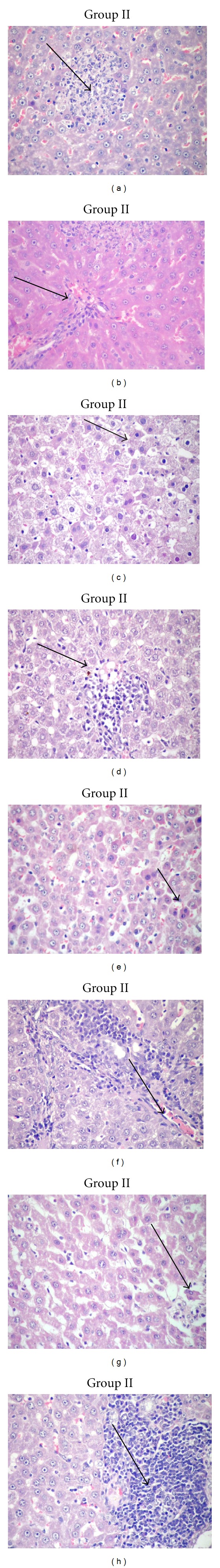
((a) and (b)) Light microscopic observations on the histological liver structures of two individual rats from induced toxicity group (group II (a) and (b)). ((c) and (d)) Light microscopic observations on the histological liver structures of two individual rats from induced toxicity group (group II (c) and (d)). ((e) and (f)) Light microscopic observations on the histological liver structures of two individual rats from induced toxicity group (group II (e) and (f)). ((g) and (h)) Light microscopic observations on the histological liver structures of two individual rats from induced toxicity group (group II (g) and (h)).

**Figure 8:**
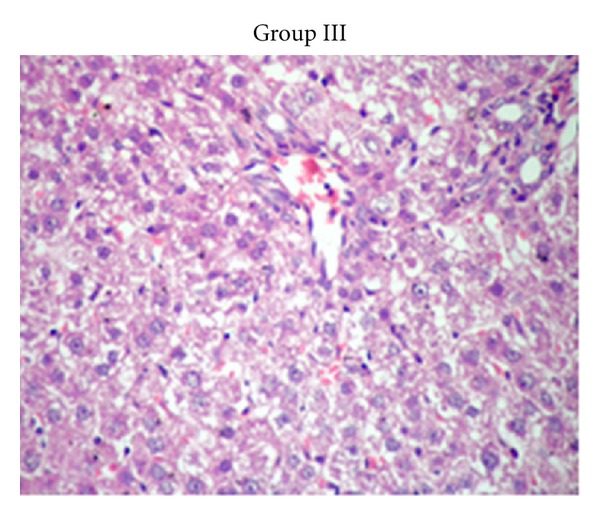
The liver showing normal hepatic architecture (H&E 400x).

**Figure 9:**
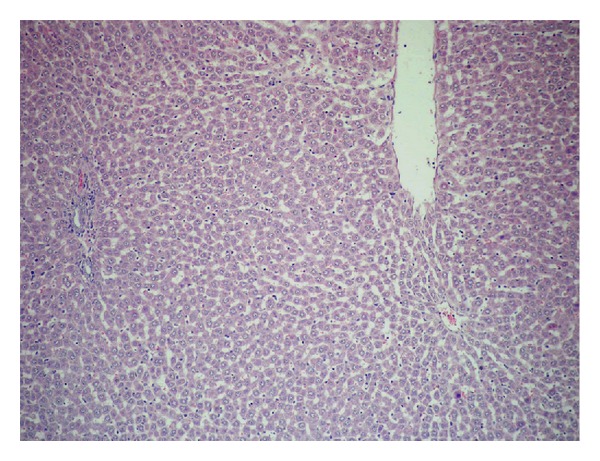
The liver showing preserved hepatic architecture with, mild portal inflammatory infiltrate and frequent apoptotic figures, group IV. (H&E 200x).

The results of rats liver histopathological studies are shown in Figures 6, 7(a)–7(h), 8 and 9 for the induced toxicity group (Group II) compared to other groups (I, III, and IV). The figures showed that group I (−ve control), and group III showed normal liver with no remarkable pathological changes in (Figures 6 GI and 8 GIII). The rat's liver of individual induced toxicity group, group II (treated with the heavy metal mixture, 0.25 mg/100 gm b.wt/7 day), showed different proportional to histopathological changes appeared as an extensive loss of hepatic architecture and large amount of damage as shown in Figures 7(a)–7(h) and summarized in Table 1. 

Some animals with the group IV, protected group (treated with fungal extract prior to their treatment by the heavy metal mixture), showed normal hepatic architecture, while others showed preserved hepatic architecture with mild portal inflammatory infiltrate and frequent apoptotic (Figure 9 G IV).

*The Effect of Trichurus Spiralis Extract on the Activities of Alanine Aminotransferase (ALT) and Aspartate Aminotransferase (AST) in the Serum of Rats, Treated with Heavy Metal Mixture (Induced Toxicity)*

**Figure 10:**
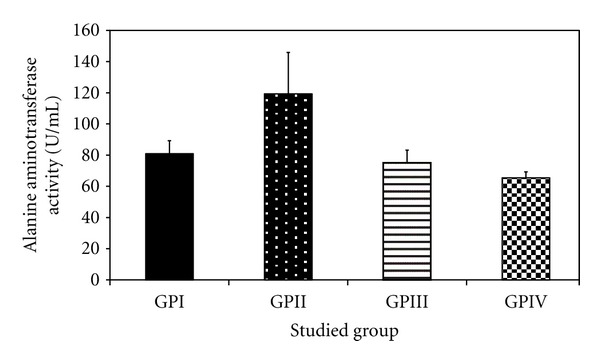
Effect of *Trichurus spiralis* extract on the activity of alanine aminotransferase (ALT, mean ± SD) in rat sera of induced toxicity group comparing to other groups.

**Figure 11:**
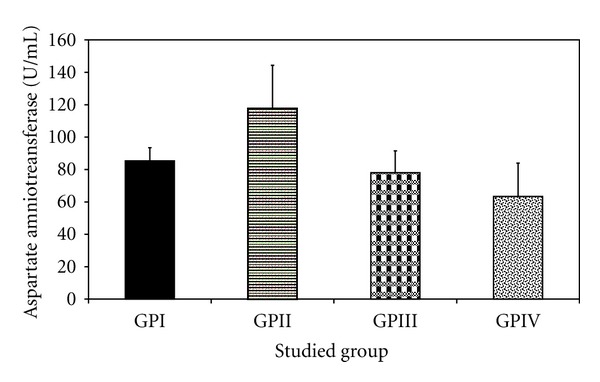
Effect of *Trichurus spiralis* extract on the activity of aspartate aminotransferase (AST, mean ± SD) in rat sera of induced toxicity group comparing to other groups.

Levels of both ALT and AST in the group IV showed nonsignificantly increase and /or decrease when compared to their corresponding values either of group, I or group III (positive control group), as shown in Table 2 and Figures 10 and 11, respectively. 

 In rats treated with the heavy metal mixture in group II, the activity of serum ALT (119.28 ± 26.58 U/L, *P* < 0.001) and AST (117.79 ± 26.58 U/L, *P* = 0.003) were significantly increased than that of −ve control group (group I) rats (85.32 ± 8.16), respectively. In contrast, the fungus extract pretreated group (group IV) at 40 mg/100 g b.wt/day for 7 days had a significantly lower ALT (65.45 ± 3.85) and AST (63.37 ± 20.54), when compared to group II, at *P* < 0.003. 

*The Effect of Trichurus Spiralis Extract on the Levels of Total Protein, Albumin and Bilirubin*

The levels of total protein and total bilirubin are found to be significantly increased in the heavy metal mixture treated group (group II) comparing to their corresponding values of –ve control group (group I) by about 38.9% and 20 times, respectively. The albumin value in group II showed a nonsignificant decrease by 14.16% compared to group I (−ve control group).

Administration of fungus extract prior to heavy metal mixture injection (group IV) showed a significant decrease in total bilirubin, compared to group II by 87.3% (0.12 ± 0.05 versus 0.95 ± 0.5 g/dL, *P* < 0.001), while the level of total protein shows a non-significant decrease compared to −ve control group. Also it showed a nonsignificant increase in albumin level between group IV and group I. 

**Figure 12:**
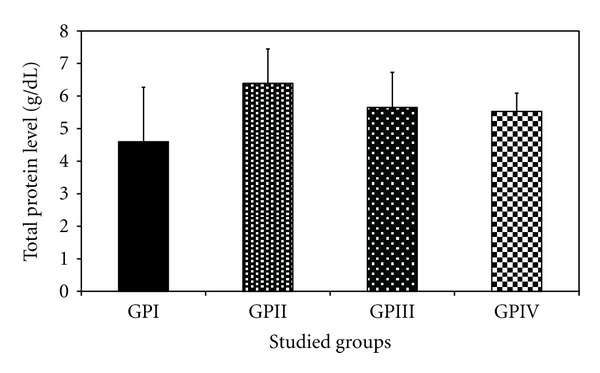
Effect of *Trichurus spiralis* extract on the level of total protein (mean ± SD) in rat sera of induced toxicity group compared to other groups.

**Figure 13:**
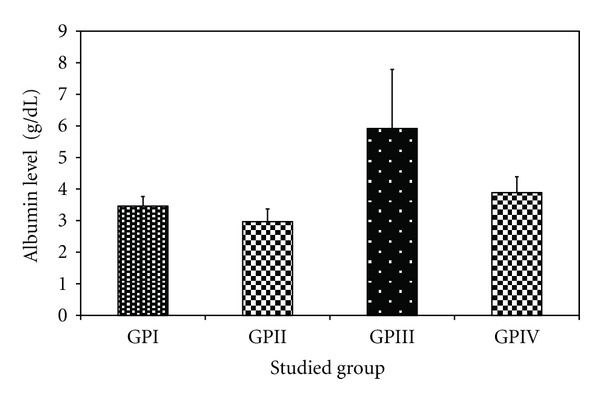
Effect of *Trichurus spiralis* extract on the level of albumin (mean ± SD) in rat sera of induced toxicity group compared to other groups.

**Figure 14:**
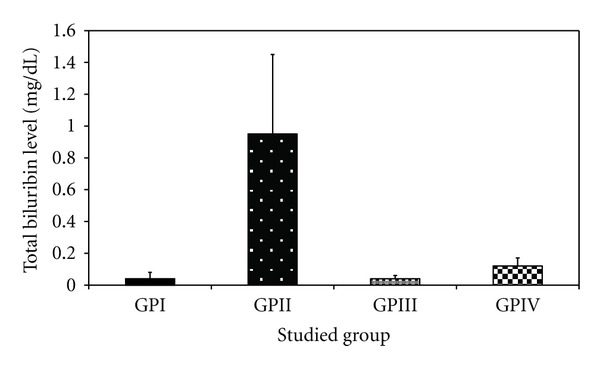
Effect of *Trichurus spiralis* extract on the level of total bilirubin (mean ± SD) in rat sera of induced toxicity group compared to other groups.

The levels of total protein, total bilirubin and albumin in group I, III, and IV showed a non-significant increase and/or decrease, when compared to each other, at *P* < 0.05, as shown in Table 3 and Figures 12, 13, and 14, respectively.

## 4. Discussion 

Liver damage mainly occurs due to excessive alcohol consumption, viral infections; and as a consequence of drug adverse effects. Nowadays, liver diseases constitute a major medical problem of worldwide proportions [77, 78].

There are approximately 35 heavy metals in our environment. Heavy metals become toxic when they are not metabolized, which allows them to accumulate in several organs leading to tissue damage due to their toxicity [79, 80]. According to ASTDR (2005–2007), the most known pollutants in our environment are Cd, Co, Hg, Ni, and Pb. On the other hand, liver tissues are the factory of biological metabolism in mammals and act as the master player in the detoxification process. Liver tissues are also a victim for heavy metal toxicity. So, our *in vivo* study are designed to investigate the hepatoprotective effect of *trichuris* extract isolated from the marine sponge, against the heavy-metal mixture of Cd, Co, Hg, Ni chloride, and Pb acetate.

Hepatic system is the major organ system involved in metabolism, detoxification, and excretion of various endogenous and exogenously administered/ingested substances, like xenobiotics, pollutants, and so forth [81]. The physiological activity in the liver results is due to to the generation of highly reactive free radicals; which covalently bonds with membrane lipids, causing lipid preoxidation. Lipid preoxidation alters the membrane permeability and causes tissue damage [81]. Since the liver is involved in various biochemical reactions; it is prone to be attacked by the free radicals and cell necrosis resulted. However, inbuilt antioxidant systems like superoxide dismutase (SOD), reduced glutathione (GSH), and so forth protect the tissue from free radical attack [12]. Excessive release of ROS powers over this system resulted in organ damage. Strengthening of inbuilt protective mechanisms or exogenous administration of antioxidants may be useful in the protection of the organs from ROS damage. In spite of phenomenal growth of the allopathic system of medicine, synthetic antioxidant/organs protectants are not available. Hence, researchers worldwide are engaged in searching for organ protective, such as hepatoprotectants drugs from natural origin [82–84].

Natural products are of considerable importance for the discovery of new therapeutic agents [85]. Apart from plants, bacteria and fungi are the most important producers of such compounds [86]. For a long time neglected as a group of producers of natural products, marine microorganisms have more recently been isolated from a variety of marine habitats such as sea water, sediments, algae, and different animals, to discover new natural products [87, 88]. In particular, sponges which are filter feeders and accumulate high numbers of microorganisms have attracted attention [89, 90]. Consistently, fungi isolated from sponges account for the highest number (28%) of novel compounds reported from marine isolates of fungi [45]. Marine isolates of fungi evidently are a rich source of chemically diverse natural products, which have not been consequently exploited so far [91]. 

 Fungi, like all living organisms, have evolved a set of mechanisms that control and respond to the uptake and accumulation of heavy metals. Possible interactions between toxic metals and fungi include: (a) production and secretion of organic acids, polysaccharides, melanin, or proteins and subsequent binding/complexion and/or precipitation of metal ions [47, 92–94], (b) metal binding to cell walls [95], (c) transport of metal cations [95–97], (d) chemical transformation of metals [47], (e) organelle compartmentation [95–97], and (f) synthesis of thiol-containing compounds, such as the non-proteinacious glutathione, phytochelatins, and the metallothioneins proteins of families 8–13 (fungi I–VI MTs), which can sequester metal ions [47, 98–101]. Among microorganisms, fungi biomass is known to possess excellent metal-binding properties which offer the advantages of having the high percentage of cell wall material [102]. 

Metal-induced toxicity is very well reported in literatures [6, 12]. These metals generate reactive species, which in turn may cause neurotoxicity, hepatotoxicity and nephrotoxicity in humans and animals [8, 103]. The aminotransferase are intracellular enzymes, which are active in the operating the reversible exchange of amino acids between alpha-amino and alph-keto acids. As all the naturally occurring amino acids can undergo aminotransferase reaction, this class of intracellular enzyme (aminotransferases) represent an important link between protein and carbohydrate's metabolism. It is now well authorized that the liver has an important function through the regulation of trace element metabolism [104, 105]. Further trace elements serve as cofactors for many enzymes in numerous metabolic pathways, therefore, changes in the distribution of these essential and toxicological consequences with regard to the metabolism of other metals [105, 106]. Those metals which are essential for maintenance of the structural and functional integrality of the living organisms are found in all living systems and are conserved within strict concentration limits in the systems [107].

However, imbalance in the supply of any of these essential elements in the body can have both nutritional and toxicological consequences with regard to the metabolism of other metals. They can further be responsible for the development of clinical signs of trace elements deficiencies or can modify the susceptibility to metal toxicity [108]. It will insinuate that metals which have similar chemical, and physical properties, would often interact biologically and antagonize or embellish each other's function [109]. That is also confirmed in our study on the activity of aminotransferases, total protein, albumin, and bilirubin, which is confirmed by histopathology of liver as shown in Tables 2 and 3 and Figures 10, 11, 12, 13, and 14.

ALT and AST in this study show a significant increase as the consequence of heavy-metal administration in group II (induction group) by 46.20% and 45.12%, respectively. This result in agreement with previous studied, which carried on exposure to mercury chloride [110–114], and Ni [115]. Furthermore, this agrees with several investigators which reported that Cd and Pd administration increase aminotransferase, especially ALT, as a result of the necrotic lesion in the liver [114, 116, 117]. As seen in Figure 7(b), on the other hand, lead overloads stimulated oxidative damage in the liver tissue by causing oxidation of lipid. These enzymes cause liver injury [118]; this was confirmed in this study by the histopatholgical result of liver tissue induced by the heavy metal mixture alone (group II) as seen in Figures 7(a)–7(h). 

Total protein, is elevated by about 38.9% in blood serum induction group compared to control group (−ve group). This observation may be as a result of the injury inflicted on the liver; thereby making the proteins synthesized in the liver and spill out into the blood [116]. Also, this result is compatible with [119, 120]. Possible explanation for protein elevation is due to toxic insult of mercury that leads to induce a number of stress proteins [119, 120]. These large groups of proteins include heat shock proteins (HSPs) and glucose regulated proteins (GRPs). As reported in [120], an enhanced *de novo* synthesis of several stress proteins when chick embryos, were exposed to mercury.

In contrast, albumin and protein are predominately produced within the liver, decreased as a consequence of metal elevation. This suggests that the heavy metals, like cadmium and lead, occurs when present in toxic concentrations in the system, impair the protein synthesis in liver [116]. 

Bilirubin is also regarded as a member of an antioxidant family even if it is known to have toxic effects at high concentration [121, 122]. Bilirubin has been regarded for many years as cytotoxic, mainly because of its associations with neonate jaundice at high concentrations [123]. It is only since the early 1990s that a physiological role for bilirubin as potent antioxidant has emerged. Reference [124] noted that bilirubin possesses strong antioxidant potential against peroxyl radicals. However, high level bilirubin may exacerbate oxidative stress [122]. Reference [125] showed that the increase of bilirubin formation due to activation of HO-2 (constitutive isoform of HO) protects against hydrogen-peroxide-induced neurotoxicity. It has been also demonstrated that intracellular bilirubin concentrations can be locally and temporarily increased by induction of HO-1 (inducible isoform of HO) or rapid activation of HO-2, so as to resist short- and long-lasting oxidative stress [123].

It has been proposed that the specific induction of HO-1 by various forms of oxidative stress, for example, different heavy metals, CCL4, and aminoacetophenone, was part of the defensive mechanism mounted by cells against stress injury, to decrease the levels of potential pro-oxidants and to increase the concentrations of active bile pigments that can act as antioxidants [126, 127]. HO-1 upregulation is followed by an increased bilirubin production, altogether determining the adaptive response of cells to oxidative stress [126].

Some natural antioxidant products have been shown to protect cells from oxidative injury [128], the high antioxidant capacity of fungal extract as shown in Figure 4, due to its high total phenolic and flavonoid content shown in Figure 3, which is confirmed *in vitro*. From the *in vitro* results, the *Trichurus* extract has high flavonoid content, where flavonoids are best known for their antioxidant properties and may act *in vitro* as reducing agents, hydrogen donors, free radical quenchers, and metal ion chelators [129].

It has been demonstrated previously that fungi, as well as algae are potentially biosorbent, for heavy metals [130–132]. This fact has also been confirmed in the present study.

According to [133], this general chelating ability of phenolic compounds is probably related to the high nucleophilic character of the aromatic rings, rather than to be specific chelating groups within the molecule. This agrees with our results, as the extract shows high total polyphenol content (phenolic and flavonoid content) as shown in Figure 3. There is another mechanism underlying their antioxidant ability. Metal ions decompose lipid hydroperoxide (LOOH) by the hemolytic cleavage at the O–O bond and give lipid alkoxyl radicals, which initiate free radical chain oxidation. Phenolic antioxidants inhibit lipid peroxidation by trapping the lipid alkoxyl radical; and that was confirmed by our results, as the fungal extract showed a high antioxidant activity and a high inhibition ratio to lipid peroxidation *in vitro*, by a percentage of 70.80% and 85%, respectively, as shown in Figure 4. This activity depends on the structure of the molecules, the number, and position within the hydroxyl group in the molecules [134]. Many flavonoids have also been found to possess hepatoprotective activity [135].

Reference [136] show that phenolic (especially flavonoids) is able to alter peroxidation kinetics, by modifying the lipid packing order. They stabilize membranes by decreasing the membrane fluidity (in a concentration-dependent manner) and hinder the diffusion of free radicals and restrict per oxidative reaction [136, 137]. According to [138], in addition to the known protein-binding capacity of flavanols and procyanidins, they can interact with membrane phospholipids through hydrogen bonding, to the polar head groups of phospholipids. As a consequence, these compounds can be accumulated at the membranes' surface, both outside and inside the cells. 

For this activity, polyphenols possess an ideal structural chemistry and have been shown to be more effective *in vitro* than vitamins E and C on the molar basis [139]. Many beneficial pharmacological properties have been attributed to flavonoids, including antioxidant, anti-inflammatory, anticarcinogenic, chemo preventive, and cytochrome-P450-inhibitory activities [140, 141]. 

In addition to high total phenolic and flavonoid content for fungal extract, the fungal extract shows high sulfur content, where sulfur is an essential component in normal physiological function and is incorporated into amino acids, proteins, enzymes, and micronutrients [142]. Humans satisfy their nutritional needs of sulfur by consuming plants and animals [143]. The high content of sulfur due to marine chemodiversty is also heightened due to the composition of sea water, which has itself a concentration of halides in sea water of 1900 mg/L Cl^−^, 65 mg/L Br^−^, 5 × 10^−4^, and I/IO_3_^−^, which are reflected by the number of compounds incorporating these elements and sulfated compounds that can account for by the relatively high concentration of sulfur, 2700 mg/L seawater. That is confirmed in our study as shown in Figure 2 [144].

 Marine natural product in general, and especially marine fungi, can be good hepatoprotective candidates in using the heavy metal as a toxicology model. AS in the bioassay-directed searching for the hepato-protective agents from natural sources, employing the closely relevant model system to human liver toxicosis, could be an effective way to identify therapeutically applicable agents [145].

## 5. Conclusion 

In conclusion, there is a beneficial influence of the investigated fungus extract against heavy-metal mixtures-intoxicated rats. We could confirm that this extract possesses hepatoprotective property due to its proven antioxidant and free radical scavenging properties, in addition to its high sulfur content. However, other possible mechanisms such as inhibition of antioxidant enzymes, induction of oxidative stress, and the influence on different signal pathways in liver cells should not be neglected. Further investigations of these matters are warranted, particularly that of fungus extract, as well as elucidation of compounds that are responsible for such activities and their effect on liver antioxidant capacity, which should be carried out.

## Figures and Tables

**Table 1:** Livers of rats in the −ve control group showed normal histopathological appearance, where livers of rats treated with heavy metal mixtures that showed many histopathological changes are listed in the following table.

Figure	Histopathological change
(a)	Marked hepatic damage evidenced by foci of lobular necrosis with neutrophilic infiltration, adjacent to dysplastic hepatocytes, congested sinusoids, and frequent apoptotic nuclei
(b)	Marked large cell dysplasia of hepatocytes with focal necrosis and mild portal inflammatory infiltrate
(c)	Liver showing hydro pic changes in hepatocytes, and moderate portal lymphoplasmacytic infiltrate
(d)	Liver showing marked parenchymal hydro pic changes with apoptosis and adjacent regenerative hepatocytes with binucleated cells
(e)	Liver showing degenerative changes with frequent apoptotic cells at the same time evidence of beginning regeneration is seen with the appearance of binucleated cells
(f)	Section in the liver showing moderate portal lymphoplasmacytic infiltration with mild interface hepatitis. (H&E 400x)
(g)	Section in the liver showing intense heavy portal lymphoplasmacytic inflammatory infiltrate and dyspalstic changes of hepatocytes. (H&E 400x)
(h)	Liver showing coagulative necrosis and karyopickosis of the hepatocytes together with evidence of regeneration, multinucleated

**Table 2:** Effect of *Trichurus Spiralis* extract on the activities of serum alanine aminotransferase (ALT) and aspartate aminotransferase (AST).

Group	ALT (U/mL) Mean ± SD	*P*_1_	AST (U/mL) Mean ± SD	*P*_1_

I (−ve control group)	80.91 ± 8.35		85.32 ± 8.16	
II (induced group)	119.28 ± 26.58	<0.001*	117.79 ± 26.56	0.003*
III (+ve control group)	75.18 ± 8.04	0.471	78.05 ± 13.54	0.470
IV (protected group)	65.45 ± 3.85	0.060	63.37 ± 20.54	0.037*

Number of rats for each group = 7, *P*_1_: *P* value of LSD test between −ve control group and other groups.

**Table 3:** Effect of *Trichurus spiralis* extract on the serum levels of total protein, albumin, and total bilirubin of induced toxicity groups of rats.

Mean ± SD Groups	I (−ve control group)	II (Induced group)	III (+ve control group)	IV (Protected group)
Total protein (g/dL)	4.60 ± 1.67	6.39 ± 1.06	5.65 ± 1.08	5.53 ± 0.56
*P*_1_		0.008*	0.101	0.146
Albumin (g/dL)	3.46 ± 0.30	2.97 ± 0.40	5.92 ± 1.87	3.89 ± 0.50
*P*_1_		**0.369**	<0.001*	0.436
Total biluribin (g/dL)	0.04 ± 0.04	0.95 ± 0.50	0.04 ± 0.02	0.12 ± 0.05
*P*_1_		<0.001*	0.992	0.571

Number of rats for each group = 7, *P*_1_: *P* value of LSD test between control group and other groups.
